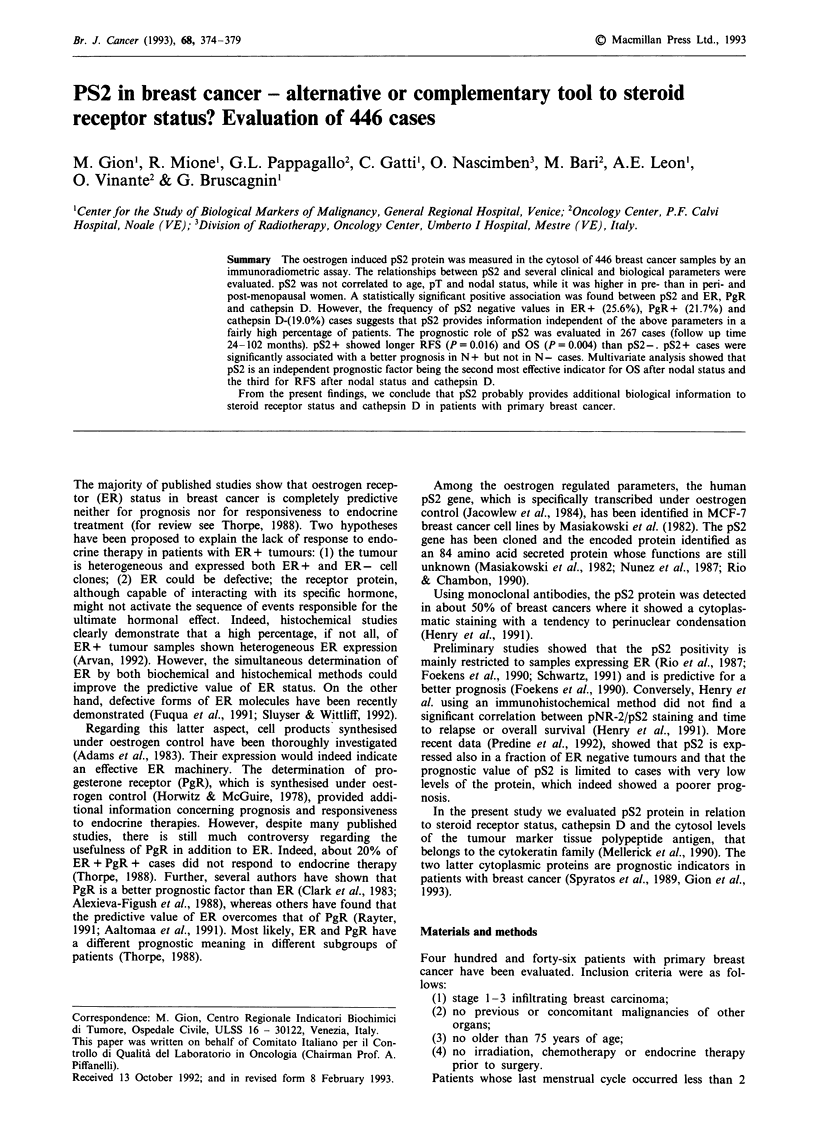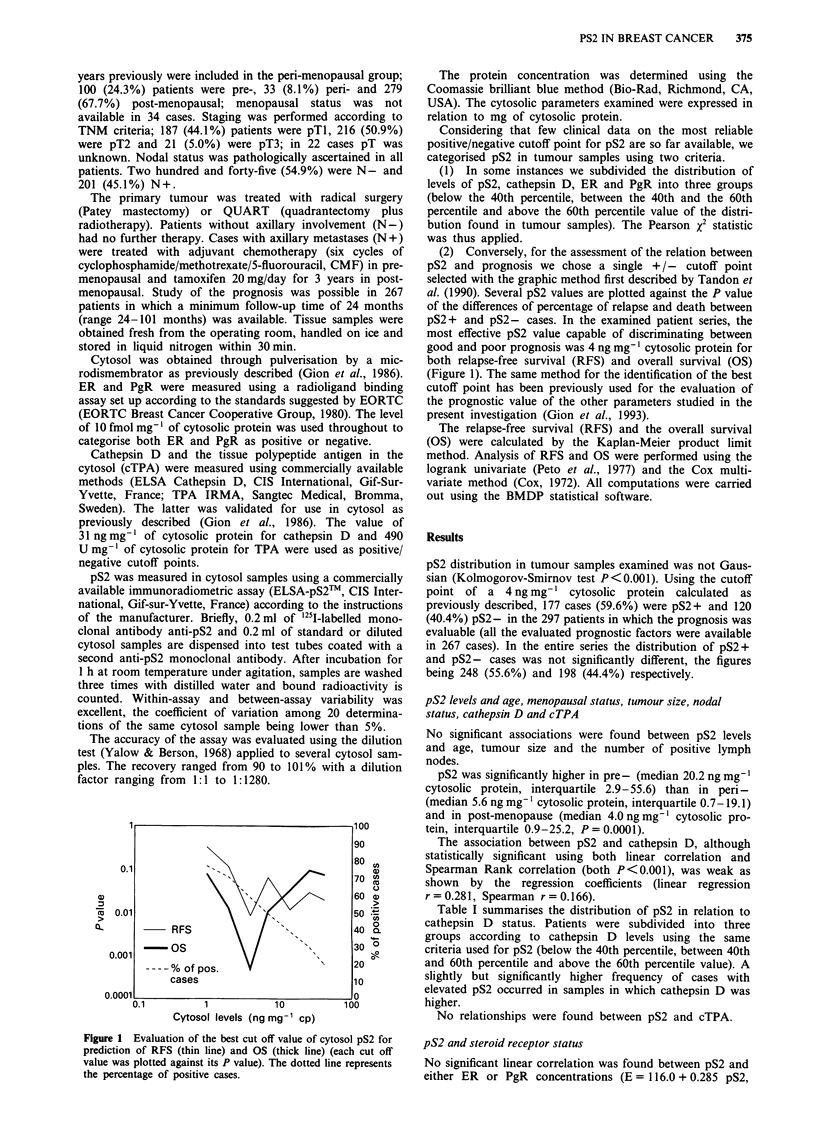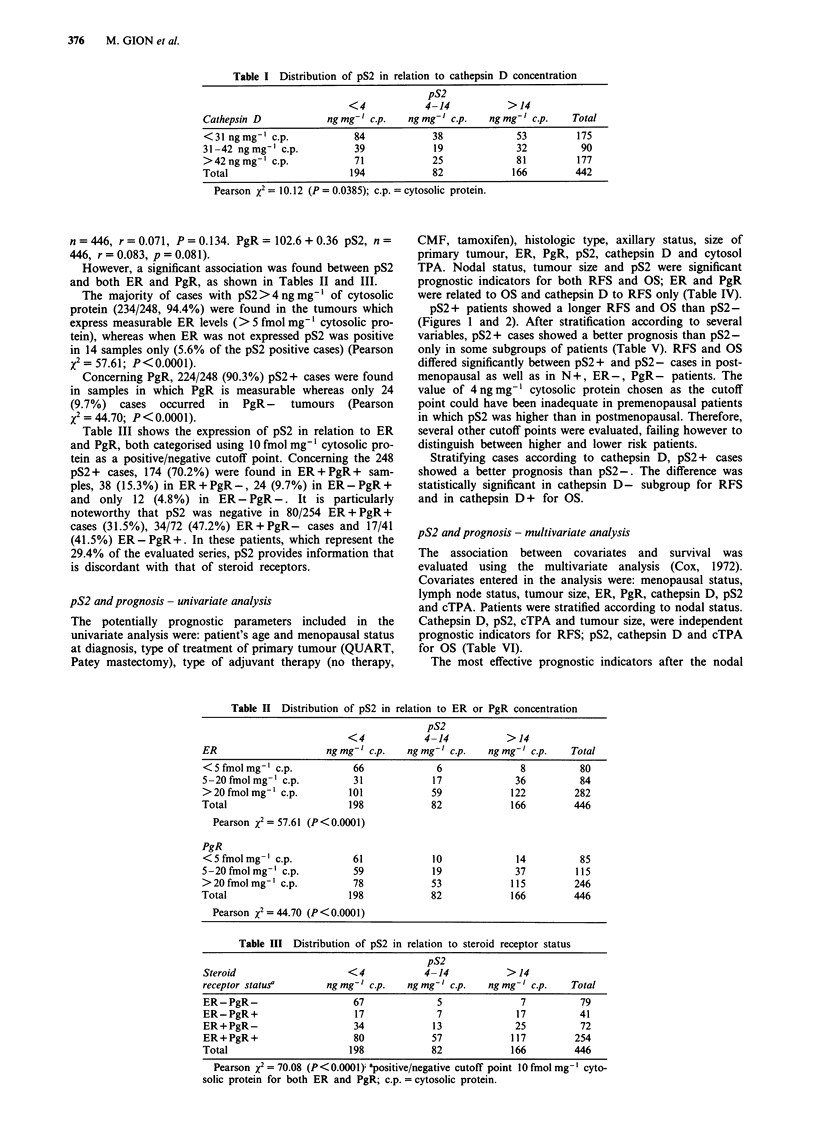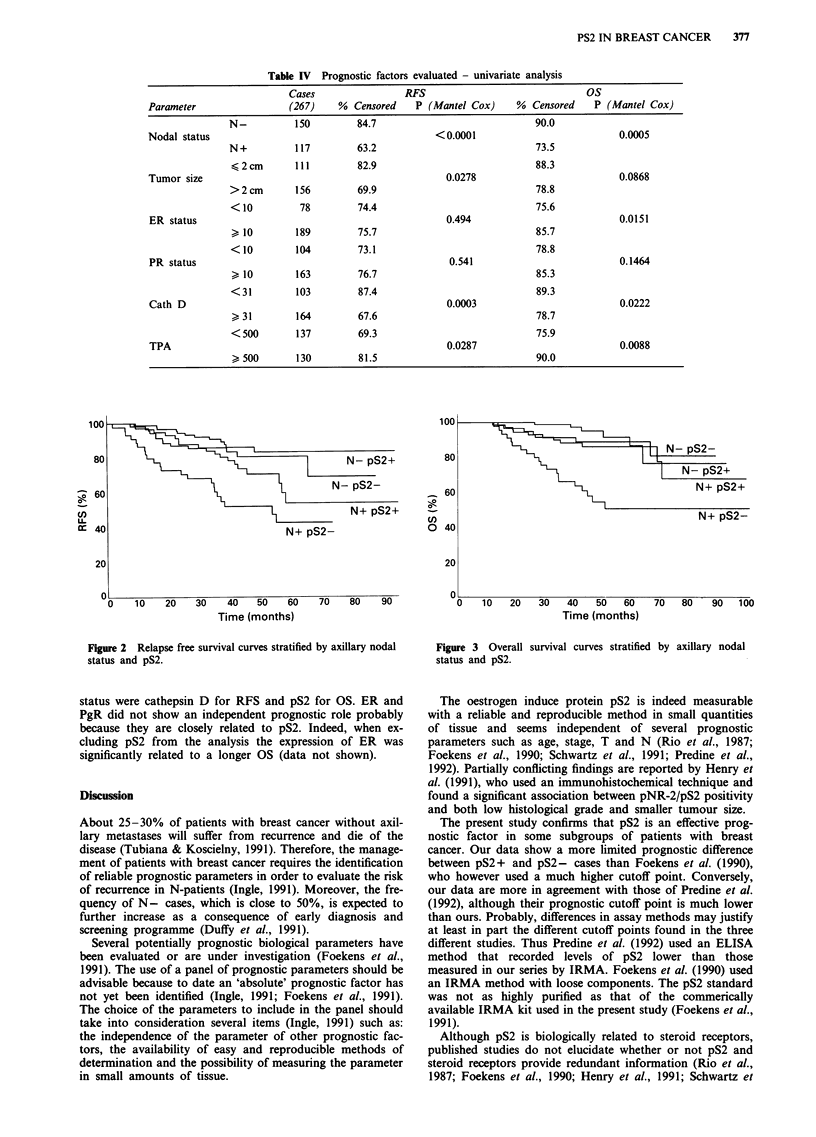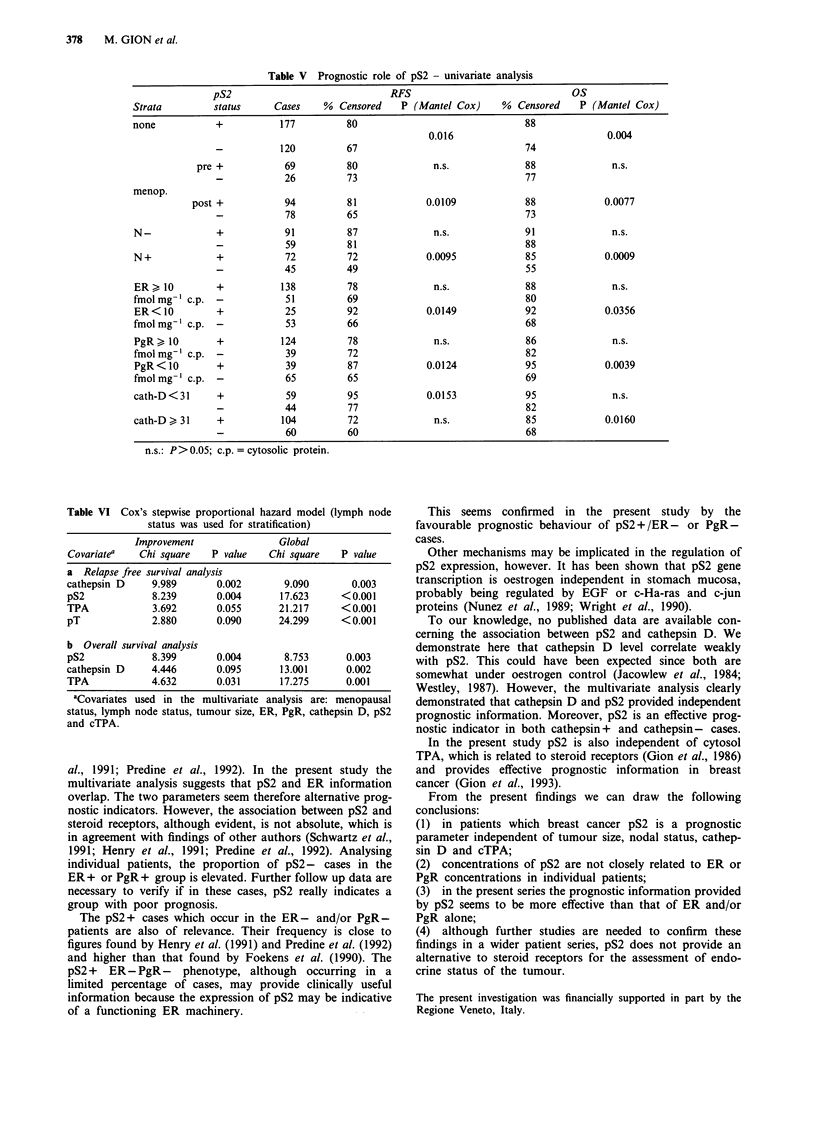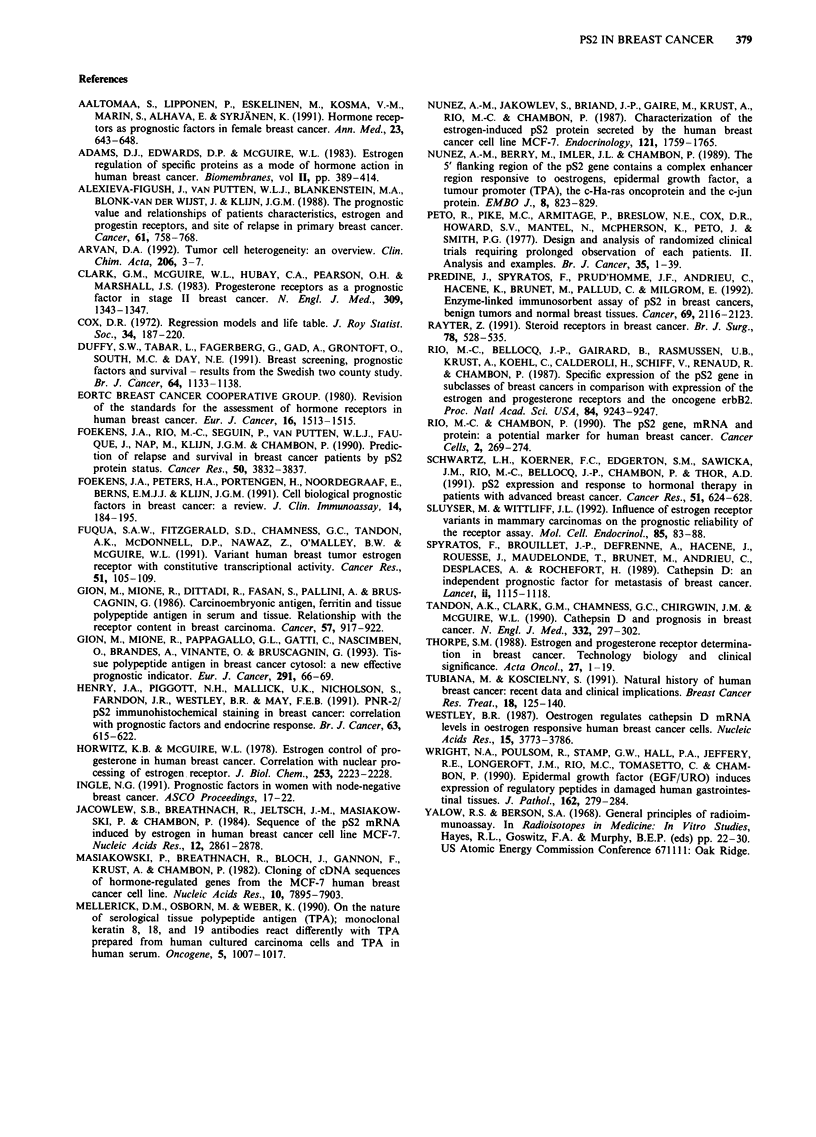# PS2 in breast cancer--alternative or complementary tool to steroid receptor status? Evaluation of 446 cases.

**DOI:** 10.1038/bjc.1993.343

**Published:** 1993-08

**Authors:** M. Gion, R. Mione, G. L. Pappagallo, C. Gatti, O. Nascimben, M. Bari, A. E. Leon, O. Vinante, G. Bruscagnin

**Affiliations:** Center for the Study of Biological Markers of Malignancy, General Regional Hospital, Venice, Italy.

## Abstract

The oestrogen induced pS2 protein was measured in the cytosol of 446 breast cancer samples by an immunoradiometric assay. The relationships between pS2 and several clinical and biological parameters were evaluated. pS2 was not correlated to age, pT and nodal status, while it was higher in pre- than in peri- and post-menopausal women. A statistically significant positive association was found between pS2 and ER, PgR and cathepsin D. However, the frequency of pS2 negative values in ER+ (25.6%), PgR+ (21.7%) and cathepsin D-(19.0%) cases suggests that pS2 provides information independent of the above parameters in a fairly high percentage of patients. The prognostic role of pS2 was evaluated in 267 cases (follow up time 24-102 months). pS2+ showed longer RFS (P = 0.016) and OS (P = 0.004) than pS2-. pS2+ cases were significantly associated with a better prognosis in N+ but not in N- cases. Multivariate analysis showed that pS2 is an independent prognostic factor being the second most effective indicator for OS after nodal status and the third for RFS after nodal status and cathepsin D. From the present findings, we conclude that pS2 probably provides additional biological information to steroid receptor status and cathepsin D in patients with primary breast cancer.


Br. J. Cancer (1993), 68, 374–379 © Macmillan Press Ltd., 1993

### PS2 in breast cancer – alternative or complementary tool to steroid receptor status? Evaluation of 446 cases

M. Gion[1], R. Mione[1], G.L. Pappagallo[2], C. Gatti[1], O. Nascimben[3], M. Bari[2], A.E. Leon[1], O. Vinante[2] & G. Bruscagnin[1]

[1]*Center for the Study of Biological Markers of Malignancy, General Regional Hospital, Venice;* [2]*Oncology Center, P.F. Calvi Hospital, Noale (VE);* [3]*Division of Radiotherapy, Oncology Center, Umberto I Hospital, Mestre (VE), Italy.*

**Summary** The oestrogen induced pS2 protein was measured in the cytosol of 446 breast cancer samples by an immunoradiometric assay. The relationships between pS2 and several clinical and biological parameters were evaluated. pS2 was not correlated to age, pT and nodal status, while it was higher in pre- than in peri- and post-menopausal women. A statistically significant positive association was found between pS2 and ER, PgR and cathepsin D. However, the frequency of pS2 negative values in ER+ (25.6%), PgR+ (21.7%) and cathepsin D-(19.0%) cases suggests that pS2 provides information independent of the above parameters in a fairly high percentage of patients. The prognostic role of pS2 was evaluated in 267 cases (follow up time 24–102 months). pS2+ showed longer RFS ($P = 0.016$) and OS ($P = 0.004$) than pS2–. pS2+ cases were significantly associated with a better prognosis in N+ but not in N– cases. Multivariate analysis showed that pS2 is an independent prognostic factor being the second most effective indicator for OS after nodal status and the third for RFS after nodal status and cathepsin D.

From the present findings, we conclude that pS2 probably provides additional biological information to steroid receptor status and cathepsin D in patients with primary breast cancer.

The majority of published studies show that oestrogen receptor (ER) status in breast cancer is completely predictive neither for prognosis nor for responsiveness to endocrine treatment (for review see Thorpe, 1988). Two hypotheses have been proposed to explain the lack of response to endocrine therapy in patients with ER+ tumours: (1) the tumour is heterogeneous and expressed both ER+ and ER– cell clones; (2) ER could be defective; the receptor protein, although capable of interacting with its specific hormone, might not activate the sequence of events responsible for the ultimate hormonal effect. Indeed, histochemical studies clearly demonstrate that a high percentage, if not all, of ER+ tumour samples shown heterogeneous ER expression (Arvan, 1992). However, the simultaneous determination of ER by both biochemical and histochemical methods could improve the predictive value of ER status. On the other hand, defective forms of ER molecules have been recently demonstrated (Fuqua *et al.*, 1991; Sluyser & Wittliff, 1992).

Regarding this latter aspect, cell products synthesised under oestrogen control have been thoroughly investigated (Adams *et al.*, 1983). Their expression would indeed indicate an effective ER machinery. The determination of progesterone receptor (PgR), which is synthesised under oestrogen control (Horwitz & McGuire, 1978), provided additional information concerning prognosis and responsiveness to endocrine therapies. However, despite many published studies, there is still much controversy regarding the usefulness of PgR in addition to ER. Indeed, about 20% of ER + PgR + cases did not respond to endocrine therapy (Thorpe, 1988). Further, several authors have shown that PgR is a better prognostic factor than ER (Clark *et al.*, 1983; Alexieva-Figush *et al.*, 1988), whereas others have found that the predictive value of ER overcomes that of PgR (Rayter, 1991; Aaltomaa *et al.*, 1991). Most likely, ER and PgR have a different prognostic meaning in different subgroups of patients (Thorpe, 1988).

Correspondence: M. Gion, Centro Regionale Indicatori Biochimici di Tumore, Ospedale Civile, ULSS 16 – 30122, Venezia, Italy.
This paper was written on behalf of Comitato Italiano per il Controllo di Qualità del Laboratorio in Oncologia (Chairman Prof. A. Piffanelli).
Received 13 October 1992; and in revised form 8 February 1993.

Among the oestrogen regulated parameters, the human pS2 gene, which is specifically transcribed under oestrogen control (Jacowlew *et al.*, 1984), has been identified in MCF-7 breast cancer cell lines by Masiakowski *et al.* (1982). The pS2 gene has been cloned and the encoded protein identified as an 84 amino acid secreted protein whose functions are still unknown (Masiakowski *et al.*, 1982; Nunez *et al.*, 1987; Rio & Chambon, 1990).

Using monoclonal antibodies, the pS2 protein was detected in about 50% of breast cancers where it showed a cytoplasmatic staining with a tendency to perinuclear condensation (Henry *et al.*, 1991).

Preliminary studies showed that the pS2 positivity is mainly restricted to samples expressing ER (Rio *et al.*, 1987; Foekens *et al.*, 1990; Schwartz, 1991) and is predictive for a better prognosis (Foekens *et al.*, 1990). Conversely, Henry *et al.* using an immunohistochemical method did not find a significant correlation between pNR-2/pS2 staining and time to relapse or overall survival (Henry *et al.*, 1991). More recent data (Predine *et al.*, 1992), showed that pS2 is expressed also in a fraction of ER negative tumours and that the prognostic value of pS2 is limited to cases with very low levels of the protein, which indeed showed a poorer prognosis.

In the present study we evaluated pS2 protein in relation to steroid receptor status, cathepsin D and the cytosol levels of the tumour marker tissue polypeptide antigen, that belongs to the cytokeratin family (Mellerick *et al.*, 1990). The two latter cytoplasmic proteins are prognostic indicators in patients with breast cancer (Spyratos *et al.*, 1989, Gion *et al.*, 1993).

### Materials and methods

Four hundred and forty-six patients with primary breast cancer have been evaluated. Inclusion criteria were as follows:

(1) stage 1–3 infiltrating breast carcinoma;
(2) no previous or concomitant malignancies of other organs;
(3) no older than 75 years of age;
(4) no irradiation, chemotherapy or endocrine therapy prior to surgery.

Patients whose last menstrual cycle occurred less than 2

years previously were included in the peri-menopausal group; 100 (24.3%) patients were pre-, 33 (8.1%) peri- and 279 (67.7%) post-menopausal; menopausal status was not available in 34 cases. Staging was performed according to TNM criteria; 187 (44.1%) patients were pT1, 216 (50.9%) were pT2 and 21 (5.0%) were pT3; in 22 cases pT was unknown. Nodal status was pathologically ascertained in all patients. Two hundred and forty-five (54.9%) were N− and 201 (45.1%) N+.

The primary tumour was treated with radical surgery (Patey mastectomy) or QUART (quadrantectomy plus radiotherapy). Patients without axillary involvement (N−) had no further therapy. Cases with axillary metastases (N+) were treated with adjuvant chemotherapy (six cycles of cyclophosphamide/methotrexate/5-fluorouracil, CMF) in pre-menopausal and tamoxifen 20 mg/day for 3 years in post-menopausal. Study of the prognosis was possible in 267 patients in which a minimum follow-up time of 24 months (range 24–101 months) was available. Tissue samples were obtained fresh from the operating room, handled on ice and stored in liquid nitrogen within 30 min.

Cytosol was obtained through pulverisation by a microdismembrator as previously described (Gion *et al.*, 1986). ER and PgR were measured using a radioligand binding assay set up according to the standards suggested by EORTC (EORTC Breast Cancer Cooperative Group, 1980). The level of 10 fmol $mg^{-1}$ of cytosolic protein was used throughout to categorise both ER and PgR as positive or negative.

Cathepsin D and the tissue polypeptide antigen in the cytosol (cTPA) were measured using commercially available methods (ELSA Cathepsin D, CIS International, Gif-Sur-Yvette, France; TPA IRMA, Sangtec Medical, Bromma, Sweden). The latter was validated for use in cytosol as previously described (Gion *et al.*, 1986). The value of 31 ng $mg^{-1}$ of cytosolic protein for cathepsin D and 490 U $mg^{-1}$ of cytosolic protein for TPA were used as positive/negative cutoff points.

pS2 was measured in cytosol samples using a commercially available immunoradiometric assay (ELSA-pS2™, CIS International, Gif-sur-Yvette, France) according to the instructions of the manufacturer. Briefly, 0.2 ml of $^{125}$I-labelled monoclonal antibody anti-pS2 and 0.2 ml of standard or diluted cytosol samples are dispensed into test tubes coated with a second anti-pS2 monoclonal antibody. After incubation for 1 h at room temperature under agitation, samples are washed three times with distilled water and bound radioactivity is counted. Within-assay and between-assay variability was excellent, the coefficient of variation among 20 determinations of the same cytosol sample being lower than 5%.

The accuracy of the assay was evaluated using the dilution test (Yalow & Berson, 1968) applied to several cytosol samples. The recovery ranged from 90 to 101% with a dilution factor ranging from 1:1 to 1:1280.

**Figure 1** Evaluation of the best cut off value of cytosol pS2 for prediction of RFS (thin line) and OS (thick line) (each cut off value was plotted against its *P* value). The dotted line represents the percentage of positive cases.

The protein concentration was determined using the Coomassie brilliant blue method (Bio-Rad, Richmond, CA, USA). The cytosolic parameters examined were expressed in relation to mg of cytosolic protein.

Considering that few clinical data on the most reliable positive/negative cutoff point for pS2 are so far available, we categorised pS2 in tumour samples using two criteria.

(1) In some instances we subdivided the distribution of levels of pS2, cathepsin D, ER and PgR into three groups (below the 40th percentile, between the 40th and the 60th percentile and above the 60th percentile value of the distribution found in tumour samples). The Pearson $\chi^2$ statistic was thus applied.

(2) Conversely, for the assessment of the relation between pS2 and prognosis we chose a single +/− cutoff point selected with the graphic method first described by Tandon *et al.* (1990). Several pS2 values are plotted against the *P* value of the differences of percentage of relapse and death between pS2+ and pS2− cases. In the examined patient series, the most effective pS2 value capable of discriminating between good and poor prognosis was 4 ng $mg^{-1}$ cytosolic protein for both relapse-free survival (RFS) and overall survival (OS) (Figure 1). The same method for the identification of the best cutoff point has been previously used for the evaluation of the prognostic value of the other parameters studied in the present investigation (Gion *et al.*, 1993).

The relapse-free survival (RFS) and the overall survival (OS) were calculated by the Kaplan-Meier product limit method. Analysis of RFS and OS were performed using the logrank univariate (Peto *et al.*, 1977) and the Cox multivariate method (Cox, 1972). All computations were carried out using the BMDP statistical software.

### Results pS2 distribution in tumour samples examined was not Gaussian (Kolmogorov-Smirnov test $P < 0.001$). Using the cutoff point of a 4 ng $mg^{-1}$ cytosolic protein calculated as previously described, 177 cases (59.6%) were pS2+ and 120 (40.4%) pS2− in the 297 patients in which the prognosis was evaluable (all the evaluated prognostic factors were available in 267 cases). In the entire series the distribution of pS2+ and pS2− cases was not significantly different, the figures being 248 (55.6%) and 198 (44.4%) respectively.

### *pS2 levels and age, menopausal status, tumour size, nodal status, cathepsin D and cTPA*

No significant associations were found between pS2 levels and age, tumour size and the number of positive lymph nodes.

pS2 was significantly higher in pre− (median 20.2 ng $mg^{-1}$ cytosolic protein, interquartile 2.9–55.6) than in peri− (median 5.6 ng $mg^{-1}$ cytosolic protein, interquartile 0.7–19.1) and in post-menopause (median 4.0 ng $mg^{-1}$ cytosolic protein, interquartile 0.9–25.2, $P = 0.0001$).

The association between pS2 and cathepsin D, although statistically significant using both linear correlation and Spearman Rank correlation (both $P < 0.001$), was weak as shown by the regression coefficients (linear regression $r = 0.281$, Spearman $r = 0.166$).

Table I summarises the distribution of pS2 in relation to cathepsin D status. Patients were subdivided into three groups according to cathepsin D levels using the same criteria used for pS2 (below the 40th percentile, between 40th and 60th percentile and above the 60th percentile value). A slightly but significantly higher frequency of cases with elevated pS2 occurred in samples in which cathepsin D was higher.

No relationships were found between pS2 and cTPA.

### *pS2 and steroid receptor status*

No significant linear correlation was found between pS2 and either ER or PgR concentrations ($E = 116.0 + 0.285$ pS2,

**Table I** Distribution of pS2 in relation to cathepsin D concentration

| *Cathepsin D* | pS2 <4 $ng\,mg^{-1}$ c.p. | pS2 4–14 $ng\,mg^{-1}$ c.p. | pS2 >14 $ng\,mg^{-1}$ c.p. | *Total* |
|---|---|---|---|---|
| <31 ng mg$^{-1}$ c.p. | 84 | 38 | 53 | 175 |
| 31–42 ng mg$^{-1}$ c.p. | 39 | 19 | 32 | 90 |
| >42 ng mg$^{-1}$ c.p. | 71 | 25 | 81 | 177 |
| Total | 194 | 82 | 166 | 442 |

Pearson $\chi^2 = 10.12$ ($P = 0.0385$); c.p. = cytosolic protein.

$n = 446$, $r = 0.071$, $P = 0.134$. PgR = 102.6 + 0.36 pS2, $n = 446$, $r = 0.083$, $p = 0.081$).

However, a significant association was found between pS2 and both ER and PgR, as shown in Tables II and III.

The majority of cases with pS2 > 4 ng mg$^{-1}$ of cytosolic protein (234/248, 94.4%) were found in the tumours which express measurable ER levels (> 5 fmol mg$^{-1}$ cytosolic protein), whereas when ER was not expressed pS2 was positive in 14 samples only (5.6% of the pS2 positive cases) (Pearson $\chi^2 = 57.61$; $P < 0.0001$).

Concerning PgR, 224/248 (90.3%) pS2+ cases were found in samples in which PgR is measurable whereas only 24 (9.7%) cases occurred in PgR− tumours (Pearson $\chi^2 = 44.70$; $P < 0.0001$).

Table III shows the expression of pS2 in relation to ER and PgR, both categorised using 10 fmol mg$^{-1}$ cytosolic protein as a positive/negative cutoff point. Concerning the 248 pS2+ cases, 174 (70.2%) were found in ER+PgR+ samples, 38 (15.3%) in ER+PgR−, 24 (9.7%) in ER−PgR+ and only 12 (4.8%) in ER−PgR−. It is particularly noteworthy that pS2 was negative in 80/254 ER+PgR+ cases (31.5%), 34/72 (47.2%) ER+PgR− cases and 17/41 (41.5%) ER−PgR+. In these patients, which represent the 29.4% of the evaluated series, pS2 provides information that is discordant with that of steroid receptors.

### *pS2 and prognosis – univariate analysis*

The potentially prognostic parameters included in the univariate analysis were: patient's age and menopausal status at diagnosis, type of treatment of primary tumour (QUART, Patey mastectomy), type of adjuvant therapy (no therapy, CMF, tamoxifen), histologic type, axillary status, size of primary tumour, ER, PgR, pS2, cathepsin D and cytosol TPA. Nodal status, tumour size and pS2 were significant prognostic indicators for both RFS and OS; ER and PgR were related to OS and cathepsin D to RFS only (Table IV).

pS2+ patients showed a longer RFS and OS than pS2− (Figures 1 and 2). After stratification according to several variables, pS2+ cases showed a better prognosis than pS2− only in some subgroups of patients (Table V). RFS and OS differed significantly between pS2+ and pS2− cases in postmenopausal as well as in N+, ER−, PgR− patients. The value of 4 ng mg$^{-1}$ cytosolic protein chosen as the cutoff point could have been inadequate in premenopausal patients in which pS2 was higher than in postmenopausal. Therefore, several other cutoff points were evaluated, failing however to distinguish between higher and lower risk patients.

Stratifying cases according to cathepsin D, pS2+ cases showed a better prognosis than pS2−. The difference was statistically significant in cathepsin D− subgroup for RFS and in cathepsin D+ for OS.

### *pS2 and prognosis – multivariate analysis*

The association between covariates and survival was evaluated using the multivariate analysis (Cox, 1972). Covariates entered in the analysis were: menopausal status, lymph node status, tumour size, ER, PgR, cathepsin D, pS2 and cTPA. Patients were stratified according to nodal status. Cathepsin D, pS2, cTPA and tumour size, were independent prognostic indicators for RFS; pS2, cathepsin D and cTPA for OS (Table VI).

The most effective prognostic indicators after the nodal

**Table II** Distribution of pS2 in relation to ER or PgR concentration

| *ER* | pS2 <4 $ng\,mg^{-1}$ c.p. | pS2 4–14 $ng\,mg^{-1}$ c.p. | pS2 >14 $ng\,mg^{-1}$ c.p. | *Total* |
|---|---|---|---|---|
| <5 fmol mg$^{-1}$ c.p. | 66 | 6 | 8 | 80 |
| 5–20 fmol mg$^{-1}$ c.p. | 31 | 17 | 36 | 84 |
| >20 fmol mg$^{-1}$ c.p. | 101 | 59 | 122 | 282 |
| Total | 198 | 82 | 166 | 446 |
| Pearson $\chi^2 = 57.61$ ($P < 0.0001$) | | | | |
| *PgR* | | | | |
| <5 fmol mg$^{-1}$ c.p. | 61 | 10 | 14 | 85 |
| 5–20 fmol mg$^{-1}$ c.p. | 59 | 19 | 37 | 115 |
| >20 fmol mg$^{-1}$ c.p. | 78 | 53 | 115 | 246 |
| Total | 198 | 82 | 166 | 446 |
| Pearson $\chi^2 = 44.70$ ($P < 0.0001$) | | | | |

**Table III** Distribution of pS2 in relation to steroid receptor status

| *Steroid receptor status*[a] | pS2 <4 $ng\,mg^{-1}$ c.p. | pS2 4–14 $ng\,mg^{-1}$ c.p. | pS2 >14 $ng\,mg^{-1}$ c.p. | *Total* |
|---|---|---|---|---|
| ER−PgR− | 67 | 5 | 7 | 79 |
| ER−PgR+ | 17 | 7 | 17 | 41 |
| ER+PgR− | 34 | 13 | 25 | 72 |
| ER+PgR+ | 80 | 57 | 117 | 254 |
| Total | 198 | 82 | 166 | 446 |

Pearson $\chi^2 = 70.08$ ($P < 0.0001$); [a]positive/negative cutoff point 10 fmol mg$^{-1}$ cytosolic protein for both ER and PgR; c.p. = cytosolic protein.

**Table IV** Prognostic factors evaluated – univariate analysis

| Parameter | | Cases (267) | RFS % Censored | RFS P (Mantel Cox) | OS % Censored | OS P (Mantel Cox) |
|---|---|---|---|---|---|---|
| Nodal status | N− | 150 | 84.7 | <0.0001 | 90.0 | 0.0005 |
| | N+ | 117 | 63.2 | | 73.5 | |
| Tumor size | ⩽2 cm | 111 | 82.9 | 0.0278 | 88.3 | 0.0868 |
| | >2 cm | 156 | 69.9 | | 78.8 | |
| ER status | <10 | 78 | 74.4 | 0.494 | 75.6 | 0.0151 |
| | ⩾10 | 189 | 75.7 | | 85.7 | |
| PR status | <10 | 104 | 73.1 | 0.541 | 78.8 | 0.1464 |
| | ⩾10 | 163 | 76.7 | | 85.3 | |
| Cath D | <31 | 103 | 87.4 | 0.0003 | 89.3 | 0.0222 |
| | ⩾31 | 164 | 67.6 | | 78.7 | |
| TPA | <500 | 137 | 69.3 | 0.0287 | 75.9 | 0.0088 |
| | ⩾500 | 130 | 81.5 | | 90.0 | |

**Figure 2** Relapse free survival curves stratified by axillary nodal status and pS2.

**Figure 3** Overall survival curves stratified by axillary nodal status and pS2.

status were cathepsin D for RFS and pS2 for OS. ER and PgR did not show an independent prognostic role probably because they are closely related to pS2. Indeed, when excluding pS2 from the analysis the expression of ER was significantly related to a longer OS (data not shown).

### Discussion

About 25–30% of patients with breast cancer without axillary metastases will suffer from recurrence and die of the disease (Tubiana & Koscielny, 1991). Therefore, the management of patients with breast cancer requires the identification of reliable prognostic parameters in order to evaluate the risk of recurrence in N-patients (Ingle, 1991). Moreover, the frequency of N− cases, which is close to 50%, is expected to further increase as a consequence of early diagnosis and screening programme (Duffy *et al.*, 1991).

Several potentially prognostic biological parameters have been evaluated or are under investigation (Foekens *et al.*, 1991). The use of a panel of prognostic parameters should be advisable because to date an 'absolute' prognostic factor has not yet been identified (Ingle, 1991; Foekens *et al.*, 1991). The choice of the parameters to include in the panel should take into consideration several items (Ingle, 1991) such as: the independence of the parameter of other prognostic factors, the availability of easy and reproducible methods of determination and the possibility of measuring the parameter in small amounts of tissue.

The oestrogen induce protein pS2 is indeed measurable with a reliable and reproducible method in small quantities of tissue and seems independent of several prognostic parameters such as age, stage, T and N (Rio *et al.*, 1987; Foekens *et al.*, 1990; Schwartz *et al.*, 1991; Predine *et al.*, 1992). Partially conflicting findings are reported by Henry *et al.* (1991), who used an immunohistochemical technique and found a significant association between pNR-2/pS2 positivity and both low histological grade and smaller tumour size.

The present study confirms that pS2 is an effective prognostic factor in some subgroups of patients with breast cancer. Our data show a more limited prognostic difference between pS2+ and pS2− cases than Foekens *et al.* (1990), who however used a much higher cutoff point. Conversely, our data are more in agreement with those of Predine *et al.* (1992), although their prognostic cutoff point is much lower than ours. Probably, differences in assay methods may justify at least in part the different cutoff points found in the three different studies. Thus Predine *et al.* (1992) used an ELISA method that recorded levels of pS2 lower than those measured in our series by IRMA. Foekens *et al.* (1990) used an IRMA method with loose components. The pS2 standard was not as highly purified as that of the commerically available IRMA kit used in the present study (Foekens *et al.*, 1991).

Although pS2 is biologically related to steroid receptors, published studies do not elucidate whether or not pS2 and steroid receptors provide redundant information (Rio *et al.*, 1987; Foekens *et al.*, 1990; Henry *et al.*, 1991; Schwartz *et*

**Table V** Prognostic role of pS2 – univariate analysis

| Strata | | pS2 status | Cases | RFS % Censored | RFS P (Mantel Cox) | OS % Censored | OS P (Mantel Cox) |
|---|---|---|---|---|---|---|---|
| none | | + | 177 | 80 | 0.016 | 88 | 0.004 |
| | | − | 120 | 67 | | 74 | |
| menop. | pre | + | 69 | 80 | n.s. | 88 | n.s. |
| | | − | 26 | 73 | | 77 | |
| | post | + | 94 | 81 | 0.0109 | 88 | 0.0077 |
| | | − | 78 | 65 | | 73 | |
| N− | | + | 91 | 87 | n.s. | 91 | n.s. |
| | | − | 59 | 81 | | 88 | |
| N+ | | + | 72 | 72 | 0.0095 | 85 | 0.0009 |
| | | − | 45 | 49 | | 55 | |
| ER ⩾ 10 fmol $mg^{-1}$ c.p. | | + | 138 | 78 | n.s. | 88 | n.s. |
| | | − | 51 | 69 | | 80 | |
| ER < 10 fmol $mg^{-1}$ c.p. | | + | 25 | 92 | 0.0149 | 92 | 0.0356 |
| | | − | 53 | 66 | | 68 | |
| PgR ⩾ 10 fmol $mg^{-1}$ c.p. | | + | 124 | 78 | n.s. | 86 | n.s. |
| | | − | 39 | 72 | | 82 | |
| PgR < 10 fmol $mg^{-1}$ c.p. | | + | 39 | 87 | 0.0124 | 95 | 0.0039 |
| | | − | 65 | 65 | | 69 | |
| cath-D < 31 | | + | 59 | 95 | 0.0153 | 95 | n.s. |
| | | − | 44 | 77 | | 82 | |
| cath-D ⩾ 31 | | + | 104 | 72 | n.s. | 85 | 0.0160 |
| | | − | 60 | 60 | | 68 | |

n.s.: $P > 0.05$; c.p. = cytosolic protein.

**Table VI** Cox's stepwise proportional hazard model (lymph node status was used for stratification)

| Covariate[a] | Improvement Chi square | P value | Global Chi square | P value |
|---|---|---|---|---|
| **a** *Relapse free survival analysis* | | | | |
| cathepsin D | 9.989 | 0.002 | 9.090 | 0.003 |
| pS2 | 8.239 | 0.004 | 17.623 | <0.001 |
| TPA | 3.692 | 0.055 | 21.217 | <0.001 |
| pT | 2.880 | 0.090 | 24.299 | <0.001 |
| **b** *Overall survival analysis* | | | | |
| pS2 | 8.399 | 0.004 | 8.753 | 0.003 |
| cathepsin D | 4.446 | 0.095 | 13.001 | 0.002 |
| TPA | 4.632 | 0.031 | 17.275 | 0.001 |

[a]Covariates used in the multivariate analysis are: menopausal status, lymph node status, tumour size, ER, PgR, cathepsin D, pS2 and cTPA.

*al.*, 1991; Predine *et al.*, 1992). In the present study the multivariate analysis suggests that pS2 and ER information overlap. The two parameters seem therefore alternative prognostic indicators. However, the association between pS2 and steroid receptors, although evident, is not absolute, which is in agreement with findings of other authors (Schwartz *et al.*, 1991; Henry *et al.*, 1991; Predine *et al.*, 1992). Analysing individual patients, the proportion of pS2− cases in the ER+ or PgR+ group is elevated. Further follow up data are necessary to verify if in these cases, pS2 really indicates a group with poor prognosis.

The pS2+ cases which occur in the ER− and/or PgR− patients are also of relevance. Their frequency is close to figures found by Henry *et al.* (1991) and Predine *et al.* (1992) and higher than that found by Foekens *et al.* (1990). The pS2+ ER−PgR− phenotype, although occurring in a limited percentage of cases, may provide clinically useful information because the expression of pS2 may be indicative of a functioning ER machinery.

This seems confirmed in the present study by the favourable prognostic behaviour of pS2+/ER− or PgR− cases.

Other mechanisms may be implicated in the regulation of pS2 expression, however. It has been shown that pS2 gene transcription is oestrogen independent in stomach mucosa, probably being regulated by EGF or c-Ha-ras and c-jun proteins (Nunez *et al.*, 1989; Wright *et al.*, 1990).

To our knowledge, no published data are available concerning the association between pS2 and cathepsin D. We demonstrate here that cathepsin D level correlate weakly with pS2. This could have been expected since both are somewhat under oestrogen control (Jacowlew *et al.*, 1984; Westley, 1987). However, the multivariate analysis clearly demonstrated that cathepsin D and pS2 provided independent prognostic information. Moreover, pS2 is an effective prognostic indicator in both cathepsin+ and cathepsin− cases.

In the present study pS2 is also independent of cytosol TPA, which is related to steroid receptors (Gion *et al.*, 1986) and provides effective prognostic information in breast cancer (Gion *et al.*, 1993).

From the present findings we can draw the following conclusions:

(1) in patients which breast cancer pS2 is a prognostic parameter independent of tumour size, nodal status, cathepsin D and cTPA;

(2) concentrations of pS2 are not closely related to ER or PgR concentrations in individual patients;

(3) in the present series the prognostic information provided by pS2 seems to be more effective than that of ER and/or PgR alone;

(4) although further studies are needed to confirm these findings in a wider patient series, pS2 does not provide an alternative to steroid receptors for the assessment of endocrine status of the tumour.

The present investigation was financially supported in part by the Regione Veneto, Italy.

### References

AALTOMAA, S., LIPPONEN, P., ESKELINEN, M., KOSMA, V.-M., MARIN, S., ALHAVA, E. & SYRJÄNEN, K. (1991). Hormone receptors as prognostic factors in female breast cancer. *Ann. Med.*, **23**, 643-648.

ADAMS, D.J., EDWARDS, D.P. & MCGUIRE, W.L. (1983). Estrogen regulation of specific proteins as a mode of hormone action in human breast cancer. *Biomembranes*, vol **II**, pp. 389-414.

ALEXIEVA-FIGUSH, J., VAN PUTTEN, W.L.J., BLANKENSTEIN, M.A., BLONK-VAN DER WIJST, J. & KLIJN, J.G.M. (1988). The prognostic value and relationships of patients characteristics, estrogen and progestin receptors, and site of relapse in primary breast cancer. *Cancer*, **61**, 758-768.

ARVAN, D.A. (1992). Tumor cell heterogeneity: an overview. *Clin. Chim. Acta*, **206**, 3-7.

CLARK, G.M., MCGUIRE, W.L., HUBAY, C.A., PEARSON, O.H. & MARSHALL, J.S. (1983). Progesterone receptors as a prognostic factor in stage II breast cancer. *N. Engl. J. Med.*, **309**, 1343-1347.

COX, D.R. (1972). Regression models and life table. *J. Roy Statist. Soc.*, **34**, 187-220.

DUFFY, S.W., TABAR, L., FAGERBERG, G., GAD, A., GRONTOFT, O., SOUTH, M.C. & DAY, N.E. (1991). Breast screening, prognostic factors and survival – results from the Swedish two county study. *Br. J. Cancer*, **64**, 1133-1138.

EORTC BREAST CANCER COOPERATIVE GROUP. (1980). Revision of the standards for the assessment of hormone receptors in human breast cancer. *Eur. J. Cancer*, **16**, 1513-1515.

FOEKENS, J.A., RIO, M.-C., SEGUIN, P., VAN PUTTEN, W.L.J., FAUQUE, J., NAP, M., KLIJN, J.G.M. & CHAMBON, P. (1990). Prediction of relapse and survival in breast cancer patients by pS2 protein status. *Cancer Res.*, **50**, 3832-3837.

FOEKENS, J.A., PETERS, H.A., PORTENGEN, H., NOORDEGRAAF, E., BERNS, E.M.J.J. & KLIJN, J.G.M. (1991). Cell biological prognostic factors in breast cancer: a review. *J. Clin. Immunoassay*, **14**, 184-195.

FUQUA, S.A.W., FITZGERALD, S.D., CHAMNESS, G.C., TANDON, A.K., MCDONNELL, D.P., NAWAZ, Z., O'MALLEY, B.W. & MCGUIRE, W.L. (1991). Variant human breast tumor estrogen receptor with constitutive transcriptional activity. *Cancer Res.*, **51**, 105-109.

GION, M., MIONE, R., DITTADI, R., FASAN, S., PALLINI, A. & BRUSCAGNIN, G. (1986). Carcinoembryonic antigen, ferritin and tissue polypeptide antigen in serum and tissue. Relationship with the receptor content in breast carcinoma. *Cancer*, **57**, 917-922.

GION, M., MIONE, R., PAPPAGALLO, G.L., GATTI, C., NASCIMBEN, O., BRANDES, A., VINANTE, O. & BRUSCAGNIN, G. (1993). Tissue polypeptide antigen in breast cancer cytosol: a new effective prognostic indicator. *Eur. J. Cancer*, **291**, 66-69.

HENRY, J.A., PIGGOTT, N.H., MALLICK, U.K., NICHOLSON, S., FARNDON, J.R., WESTLEY, B.R. & MAY, F.E.B. (1991). PNR-2/pS2 immunohistochemical staining in breast cancer: correlation with prognostic factors and endocrine response. *Br. J. Cancer*, **63**, 615-622.

HORWITZ, K.B. & MCGUIRE, W.L. (1978). Estrogen control of progesterone in human breast cancer. Correlation with nuclear processing of estrogen receptor. *J. Biol. Chem.*, **253**, 2223-2228.

INGLE, N.G. (1991). Prognostic factors in women with node-negative breast cancer. *ASCO Proceedings*, 17-22.

JACOWLEW, S.B., BREATHNACH, R., JELTSCH, J.-M., MASIAKOWSKI, P. & CHAMBON, P. (1984). Sequence of the pS2 mRNA induced by estrogen in human breast cancer cell line MCF-7. *Nucleic Acids Res.*, **12**, 2861-2878.

MASIAKOWSKI, P., BREATHNACH, R., BLOCH, J., GANNON, F., KRUST, A. & CHAMBON, P. (1982). Cloning of cDNA sequences of hormone-regulated genes from the MCF-7 human breast cancer cell line. *Nucleic Acids Res.*, **10**, 7895-7903.

MELLERICK, D.M., OSBORN, M. & WEBER, K. (1990). On the nature of serological tissue polypeptide antigen (TPA); monoclonal keratin 8, 18, and 19 antibodies react differently with TPA prepared from human cultured carcinoma cells and TPA in human serum. *Oncogene*, **5**, 1007-1017.

NUNEZ, A.-M., JAKOWLEV, S., BRIAND, J.-P., GAIRE, M., KRUST, A., RIO, M.-C. & CHAMBON, P. (1987). Characterization of the estrogen-induced pS2 protein secreted by the human breast cancer cell line MCF-7. *Endocrinology*, **121**, 1759-1765.

NUNEZ, A.-M., BERRY, M., IMLER, J.L. & CHAMBON, P. (1989). The 5' flanking region of the pS2 gene contains a complex enhancer region responsive to oestrogens, epidermal growth factor, a tumour promoter (TPA), the c-Ha-ras oncoprotein and the c-jun protein. *EMBO J.*, **8**, 823-829.

PETO, R., PIKE, M.C., ARMITAGE, P., BRESLOW, N.E., COX, D.R., HOWARD, S.V., MANTEL, N., MCPHERSON, K., PETO, J. & SMITH, P.G. (1977). Design and analysis of randomized clinical trials requiring prolonged observation of each patients. II. Analysis and examples. *Br. J. Cancer*, **35**, 1-39.

PREDINE, J., SPYRATOS, F., PRUD'HOMME, J.F., ANDRIEU, C., HACENE, K., BRUNET, M., PALLUD, C. & MILGROM, E. (1992). Enzyme-linked immunosorbent assay of pS2 in breast cancers, benign tumors and normal breast tissues. *Cancer*, **69**, 2116-2123.

RAYTER, Z. (1991). Steroid receptors in breast cancer. *Br. J. Surg.*, **78**, 528-535.

RIO, M.-C., BELLOCQ, J.-P., GAIRARD, B., RASMUSSEN, U.B., KRUST, A., KOEHL, C., CALDEROLI, H., SCHIFF, V., RENAUD, R. & CHAMBON, P. (1987). Specific expression of the pS2 gene in subclasses of breast cancers in comparison with expression of the estrogen and progesterone receptors and the oncogene erbB2. *Proc. Natl Acad. Sci. USA*, **84**, 9243-9247.

RIO, M.-C. & CHAMBON, P. (1990). The pS2 gene, mRNA and protein: a potential marker for human breast cancer. *Cancer Cells*, **2**, 269-274.

SCHWARTZ, L.H., KOERNER, F.C., EDGERTON, S.M., SAWICKA, J.M., RIO, M.-C., BELLOCQ, J.-P., CHAMBON, P. & THOR, A.D. (1991). pS2 expression and response to hormonal therapy in patients with advanced breast cancer. *Cancer Res.*, **51**, 624-628.

SLUYSER, M. & WITTLIFF, J.L. (1992). Influence of estrogen receptor variants in mammary carcinomas on the prognostic reliability of the receptor assay. *Mol. Cell. Endocrinol.*, **85**, 83-88.

SPYRATOS, F., BROUILLET, J.-P., DEFRENNE, A., HACENE, J., ROUESSE, J., MAUDELONDE, T., BRUNET, M., ANDRIEU, C., DESPLACES, A. & ROCHEFORT, H. (1989). Cathepsin D: an independent prognostic factor for metastasis of breast cancer. *Lancet*, **ii**, 1115-1118.

TANDON, A.K., CLARK, G.M., CHAMNESS, G.C., CHIRGWIN, J.M. & MCGUIRE, W.L. (1990). Cathepsin D and prognosis in breast cancer. *N. Engl. J. Med.*, **332**, 297-302.

THORPE, S.M. (1988). Estrogen and progesterone receptor determination in breast cancer. Technology biology and clinical significance. *Acta Oncol.*, **27**, 1-19.

TUBIANA, M. & KOSCIELNY, S. (1991). Natural history of human breast cancer: recent data and clinical implications. *Breast Cancer Res. Treat.*, **18**, 125-140.

WESTLEY, B.R. (1987). Oestrogen regulates cathepsin D mRNA levels in oestrogen responsive human breast cancer cells. *Nucleic Acids Res.*, **15**, 3773-3786.

WRIGHT, N.A., POULSOM, R., STAMP, G.W., HALL, P.A., JEFFERY, R.E., LONGEROFT, J.M., RIO, M.C., TOMASETTO, C. & CHAMBON, P. (1990). Epidermal growth factor (EGF/URO) induces expression of regulatory peptides in damaged human gastrointestinal tissues. *J. Pathol.*, **162**, 279-284.

YALOW, R.S. & BERSON, S.A. (1968). General principles of radioimmunoassay. In *Radioisotopes in Medicine: In Vitro Studies*, Hayes, R.L., Goswitz, F.A. & Murphy, B.E.P. (eds) pp. 22-30. US Atomic Energy Commission Conference 671111: Oak Ridge.